# Renal Cell Carcinoma with Supradiaphragmatic Tumor Thrombus: Avoiding Sternotomy and Cardiopulmonary Bypass

**DOI:** 10.18103/mra.v10i7.2913

**Published:** 2022-07-31

**Authors:** Marina M. Tabbara, Javier González, Gaetano Ciancio

**Affiliations:** 1 Department of Surgery, University of Miami Miller School of Medicine; Miami, Florida; 2 Miami Transplant Institute, University of Miami Miller School of Medicine, Jackson Memorial Hospital; Miami, Florida; 3 Servicio de Urología, Unidad de Trasplante Renal, Hospital General Universitario Gregorio Marañón; Madrid, Spain; 4 Department of Urology, University of Miami Miller School of Medicine; Miami, Florida

**Keywords:** renal cell carcinoma, tumor thrombus, nephrectomy, thrombectomy, cardiopulmonary bypass, sternotomy, surgical technique

## Abstract

Renal cell carcinoma (RCC) accounts for 2–3% of all malignant disease in adults and has a propensity to infiltrate the surrounding adjacent structures with a biologic predisposition for vascular invasion. This tropism for the venous system facilitates propagation into the renal vein and inferior vena cava (IVC) in up to 25% of patients with RCC. Surgical resection remains the mainstay treatment for RCC with venous tumor thrombus (TT) extension and the only hope for a potential cure. Higher thrombus levels correlate with more advanced stages of disease and thus poorer survival rates. Although CPB with circulatory arrest has been successfully performed during resection of these tumors, its use remains controversial due to the risk of coagulopathy, platelet dysfunction, and central nervous system complications. Complete intraabdominal surgical excision of level III thrombi can be achieved without sternotomy and CPB by utilizing hepatic mobilization maneuvers. The purpose of this review is to provide an update on the surgical management of these difficult cases of RCC with supradiaphragmatic tumor thrombi, including a description of transplant-based techniques that avoid sternotomy and cardiopulmonary bypass (CPB), minimizing intra- and post-operative complications.

## INTRODUCTION

Renal cell carcinoma (RCC) represents 3% of all adult malignancies, with 30% of RCC diagnosed at locally advanced or metastatic stages of disease.^1,2^ A special form of locally advanced disease is the tumor thrombus (TT), a neoplastic extension of the tumor inside the vein due to the unique proclivity of RCC to involve vascular structures. TT commonly grows from the intrarenal veins, through the main renal vein, and up the inferior vena cava (IVC) and rarely, the right cardiac chambers. TT extension into the IVC occurs in up to 10% of cases of RCC, reaching up to the right atrium in about 1% of cases.^1–7^

RCC is rather insensitive to classic chemo- or radiotherapy protocols, therefore surgery is the only therapeutic approach with curative intention. Overall survival rates of 68% at 5 years have been reported in the best surgical candidates (absence of distant metastatic spreading in cross-sectional imaging at debut); meaning that the presence of TT itself does not imply a worse prognosis.^8^ However, it seems reasonable that higher thrombus levels correlate with more advanced stages of disease and thus poorer survival rates.^9,10^ The objective of surgery is the complete removal of neoplastic tissue, including the intravascular component, which is often the most difficult part of the intervention. Complications are rather frequent and can be devastating, making the procedure a challenge for both the surgeon and patient.

The purpose of this review is to provide an update on the surgical management of these difficult cases of RCC with supradiaphragmatic tumor thrombi, including a description of specific techniques used to minimize intra- and post-operative complications by avoiding sternotomy and cardiopulmonary bypass (CPB).

### Tumor thrombus anatomic level and classification systems

**Figure 1.**
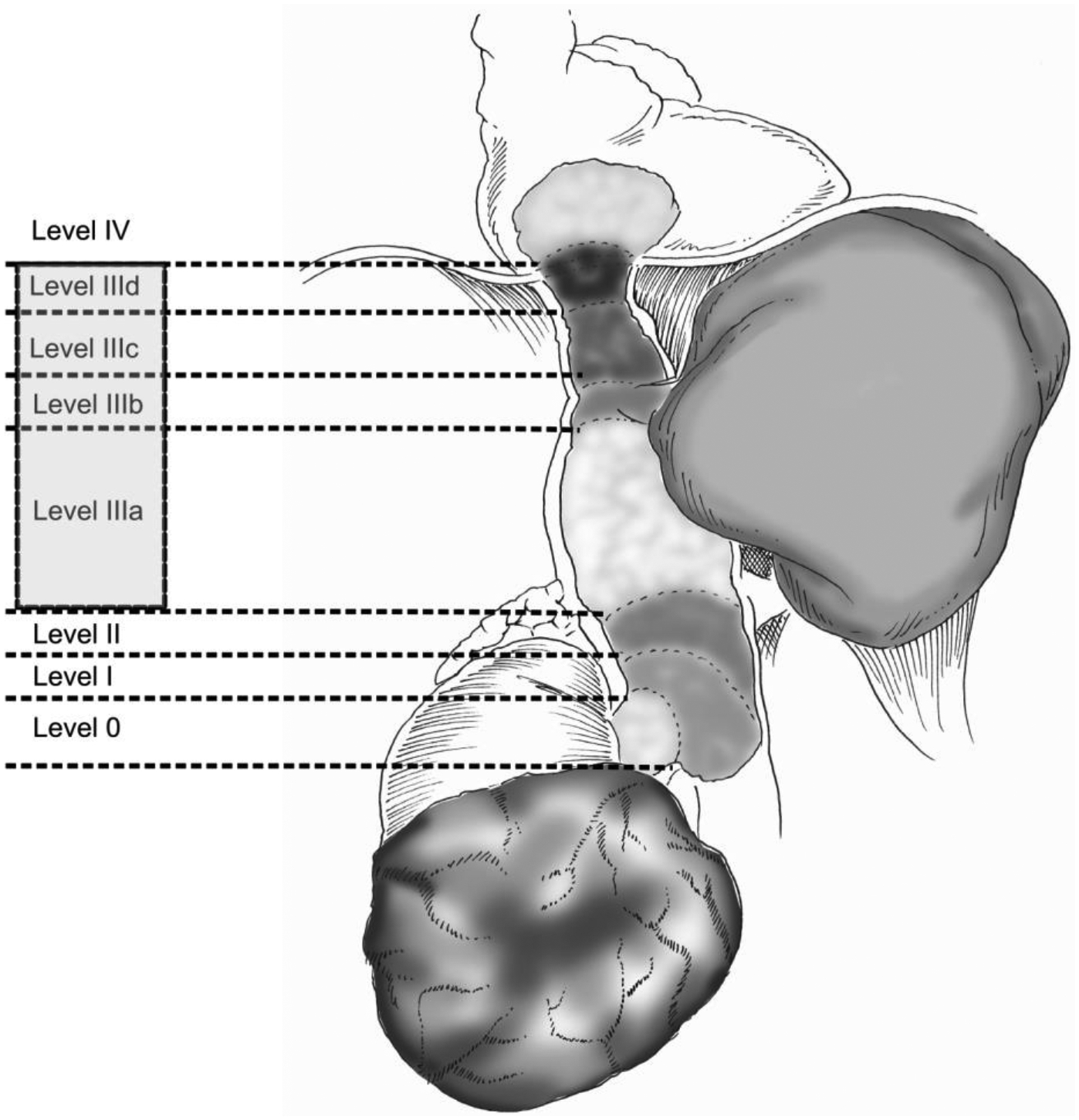
A combination of the Neves-Zincke and University of Miami Classification Systems for the tumor thrombus location inside the inferior vena cava lumen. The University of Miami Classification System is divided into four categories: IIIa (retrohepatic IVC below major hepatic veins), IIIb (retrohepatic IVC reaching the ostia of major hepatic veins), IIIc (retrohepatic IVC and extending above major hepatic veins, but below diaphragm), and IIId (suprahepatic and supradiaphragmatic IVC, reaching intrapericardial IVC, but infra-atrial).

A crucial element in preoperative workup is to determine the level of the tumor thrombus. Many classification systems have been used in the last 50 years to classify these types of tumors, providing not only prognostic information but practical information for surgical planning. The most commonly used classification system is the one described by Neves and Zincke.^11^ As dealing with higher level thrombi become increasingly more complicated, authors at the University of Miami Miller School of Medicine^12^ further expanded the classification for level III tumor thrombi to provide a clear depiction of the surgical maneuvers that should be used for each case. The authors subdivided level III thrombi into four groups: IIIa (retrohepatic IVC below major hepatic veins), IIIb (retrohepatic IVC reaching the ostia of major hepatic veins), IIIc (retrohepatic IVC and extending above major hepatic veins, but below diaphragm), and IIId (suprahepatic and supradiaphragmatic IVC, reaching intrapericardial IVC, but infra-atrial) (Figure 1).

### Avoiding sternotomy and cardiopulmonary bypass (CPB)

A multidisciplinary approach is critically important in the treatment of RCC patients with supradiaphragmatic thrombi as resection of the tumor thrombus can lead to complications such as venous congestion, embolic events, and excessive blood loss^13,14^. Complications depend mainly on the level of vascular involvement, venous redistribution in response to IVC, and the sequence of surgical steps performed.^13–17^

CPB with circulatory arrest has been successfully performed during resection of these tumors.^18^ Some authors prefer the use of CPB in efforts to decrease the risk of unexpected and life-threatening intraoperative hemorrhage and incomplete tumor expiration,^19^ however, its use remains controversial due to the risk of renal failure, coagulopathy, platelet dysfunction, and central nervous system complications.^20,21^ Complete intraabdominal surgical excision of level III and IV thrombi can be achieved without the sternotomy and CPB by utilizing hepatic mobilization maneuvers.^20,22–24^

In the case of level III tumor thrombi, Ciancio et al^22^ describe a transplant-based approach for gaining access to the retrohepatic IVC. The liver is mobilized by dissection and division of the ligamentum teres, followed by cautery division of the falciform ligament. The incision is then carried down to the right superior coronary and triangular ligaments. The visceral peritoneum overlying the right hepatic hilum and the infrahepatic IVC are then incised together with the right inferior coronary and hepatorenal ligaments. The liver can then be rolled to the left abdomen. Surgical control of the hepatic hilum is performed, permitting isolation and control of the porta hepatis to permit a Pringle maneuver when needed (if the thrombus extends above the hepatic veins).^15,25^ Then, a piggyback maneuver can be performed.^26^ Minor hepatic veins draining into the anterior surface of the IVC are ligated and divided, allowing the infrahepatic, retrohepatic and suprahepatic portions of the IVC to be completely exposed. Finally, the posterior surface of the IVC is completely dissected in order to obtain total circumferential dissection of the IVC.

**Figure 2.**
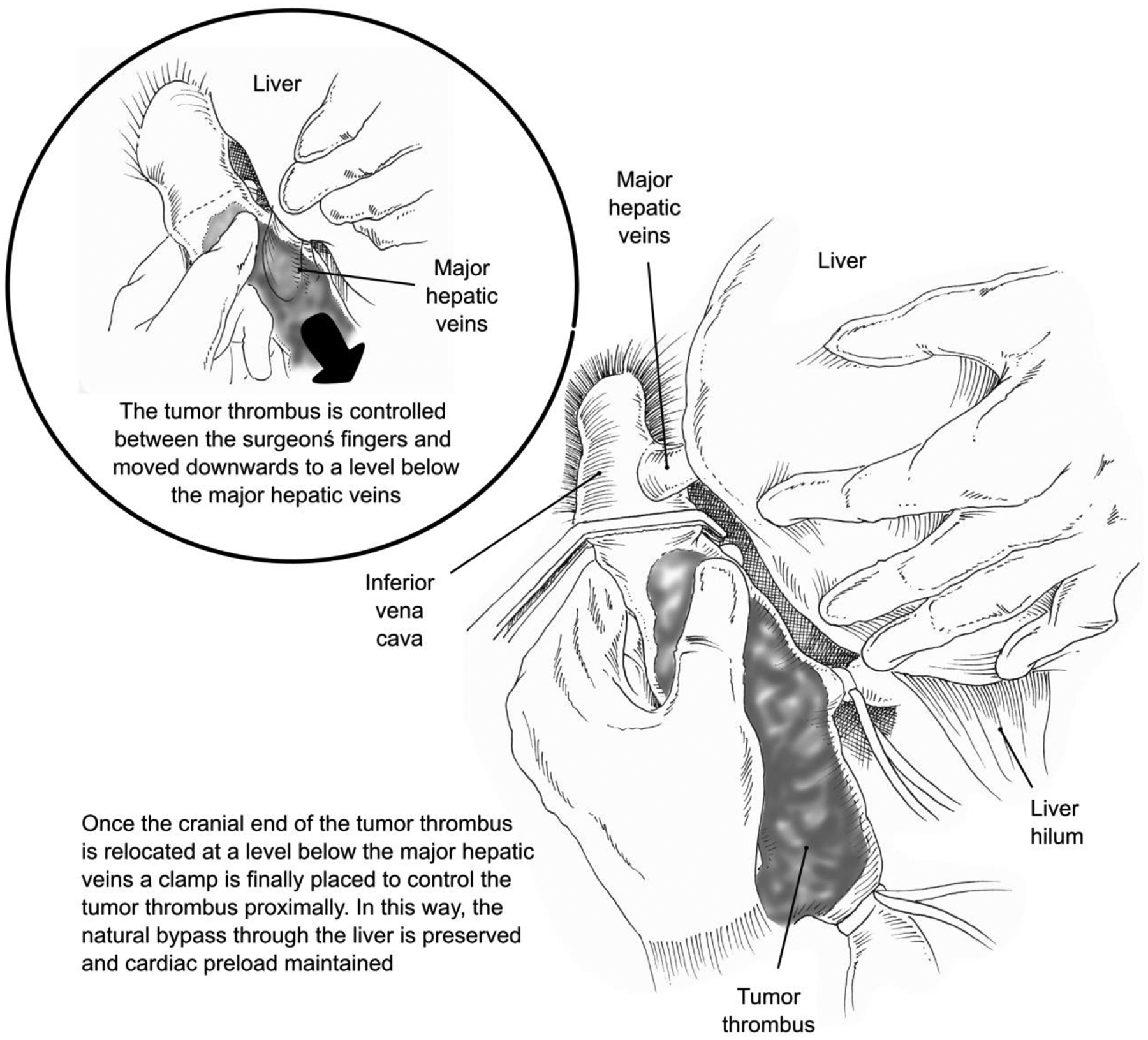
The milking maneuver.

If the TT can be milked down below the hepatic venous confluence, a clamp may be applied on IVC below the hepatic venous outflow, thereby avoiding liver congestion.^27^ This step should be monitored with TEE to assess the level of the clamp and the potential dislodgement of the thrombus and possible subsequent pulmonary embolism. This milking maneuver (Figure 2) avoids hepatic dysfunction and preserves liver drainage into the IVC through the suprahepatic veins. This technique is often feasible, especially when early ligation of renal artery is performed as blood supply to the tumor thrombus is reduced. For level IIId thrombi (suprahepatic and supradiaphragmatic IVC, reaching intrapericardial IVC, but infra-atrial), and for those cases where the milking maneuver is not feasible, dissection continues until the supradiaphragmatic and intrapericardial IVC is exposed. Intrapericadial IVC exposure requires the opening of the central tendon of the diaphragm in the midline. After gaining complete circumferential control over the IVC at this level, gentle traction at the cavo-atrial junction permits the relocation of the right atrium inside the abdomen where it can be also controlled with vascular clamps.^28^ Thrombectomy can then be performed after sequential vascular clamping as follows: i) IVC below the thrombus, ii) contralateral renal vein (and right adrenal vein in case of left-sided renal tumor), iii) Pringle maneuver, and iv) IVC above the TT (below the MHVs if milking maneuver was successful or supradiaphragmatic IVC if not). This vascular clamping is done with the help of TEE.^21,25^ However, the combination of flow interruption including the IVC and the liver circuit (Pringle maneuver) may result in hemodynamic instability due to insufficient venous return. Thus, a test clamp is recommended prior to proceeding with cavotomy. In the case of significant hypotension, either rapid infusion (i.e., fluids or blood products) through a central line or bypass instauration (i.e., veno-venous or cardiopulmonary) is recommended.^5^ However, cardiac preload is commonly guaranteed through the natural liver bypass in partially occluding lower-level thrombi. Higher-level thrombi (level IIIb-IV) exhibit larger TT diameters, occasionally invading the IVC wall. In these later cases, collateralization is the rule favoring tolerance to the combination IVC cross-clamping and Pringle maneuver.

The use of CPB with or without hypothermic circulatory arrest in management of level IV thrombi has been widely accepted.^18,19^ In cases of non-massive atrial involvement, right atrium control may be gained exclusively through the abdomen following the principles of the transplant-based approach described above, thus avoiding the need for sternotomy and CPB in most instances.^28^

In a large observational study using the International Renal Cell Carcinoma-Venous Thrombus Consortium (IRCC-VTC), Gonzalez et al^14^ compared the clinical outcomes following radical nephrectomy and tumor thrombectomy utilizing a transplant-based (TB) approach vs. a non-transplant based (non-TB) approach in patients with RCC with level II-IV thrombi. The TB approach, which required CPB in 4.1% (4/98) of cases, was superior in limiting blood loss and the development of postoperative complications (PC) when compared to a non-TB approach that required CPB in 28.1% (82/292) of cases. The percentage who developed any PC (Clavien Grade 1–5) and PC categorized by minor (Clavien Grade 1–2) and major (Clavien Grade 3–5) grades were both significantly higher among those receiving the non-TB (vs. TB) approach, with 70.5% (206/292) vs. 15.3% (15/98) developing any PC, 49.3% (144/292) vs. 7.1% (7/98) developing a minor PC, and 21.2% (62/292) vs. 8.2% (8/98) developing a major PC, respectively.

### Recent advances

In recent years, minimally invasive kidney cancer surgery for the management of radical nephrectomy and IVC tumor thrombectomy has been increasingly reported in the literature.^29–34^ Laparoscopic techniques for excision of locally invasive RCC have been described for over two decades, however its utilization for resection of higher level thrombi are considered challenging.^31^ Advances in robotic surgery and techniques have recently been applied to IVC thrombectomy.

Wang et al^35^ proposed a robotic approach focused on the spatial relationship between the TT cranial end and the location of the hepatic hilum and MHVs. This technique follows the surgical principles of the transplant-based approach, avoiding sternotomy and CPB.^25^ For patients with level IIIa TT, the Pringle maneuver was not required. For cases of level IIIb-d TT, the liver was mobilized in a piggy-back fashion and the Pringle maneuver was established before clamping the suprahepatic segment of the IVC under TEE. Cases exhibiting complete caval occlusion with sufficient collateralization underwent IVC stapling with an endo-GIA. In 2019, the authors discuss their technique when confronted with a challenging scenario of level III-IV thrombi,^36^ carefully selecting the candidate and excluding those cases in which caval invasion was anticipated. In the same way, they highlighted the importance of a multidisciplinary team in both preoperative planning and surgical intervention.

## CONCLUSIONS

Renal cell carcinoma with vascular involvement remains a surgical challenge that has lasted for more than a century. Adequate classification of the anatomical level of the TT inside the IVC has made it possible to design surgical strategies personalized to each case, obviating the need of access to cavities other than the abdomen or auxiliary procedures such as CPB for safe and successful resection.

## References

[1] Haidar GM, Hicks TD, El-Sayed HF, Davies MG. Treatment options and outcomes for caval thrombectomy and resection for renal cell carcinoma. J Vasc Surg Venous Lymphat Disord. May 2017;5(3):430–436. doi:10.1016/j.jvsv.2016.12.011 28411712

[2] Gonzalez J Update on surgical management of renal cell carcinoma with venous extension. Curr Urol Rep. Feb 2012;13(1):8–15. doi:10.1007/s11934-011-0222-0 22052458

[3] Lardas M, Stewart F, Scrimgeour D, Systematic Review of Surgical Management of Nonmetastatic Renal Cell Carcinoma with Vena Caval Thrombus. Eur Urol. Aug 2016;70(2):265–80. doi:10.1016/j.eururo.2015.11.034 26707869

[4] Calero A, Armstrong PA. Renal cell carcinoma accompanied by venous invasion and inferior vena cava thrombus: classification and operative strategies for the vascular surgeon. Semin Vasc Surg. Dec 2013;26(4):219–25. doi:10.1053/j.semvascsurg.2014.06.015 25220330

[5] Psutka SP, Leibovich BC. Management of inferior vena cava tumor thrombus in locally advanced renal cell carcinoma. Ther Adv Urol. Aug 2015;7(4):216–29. doi:10.1177/1756287215576443 26445601 PMC4580091

[6] Marshall VF, Middleton RG, Holswade GR, Goldsmith EI. Surgery for renal cell carcinoma in the vena cava. J Urol. Apr 1970;103(4):414–20. doi:10.1016/s0022-5347(17)61970-0 5437743

[7] Karnes RJ, Blute ML. Surgery insight: management of renal cell carcinoma with associated inferior vena cava thrombus. Nat Clin Pract Urol. Jun 2008;5(6):329–39. doi:10.1038/ncpuro1122 18477994

[8] Shiff B, Breau RH, Mallick R, Prognostic significance of extent of venous tumor thrombus in patients with non-metastatic renal cell carcinoma: Results from a Canadian multi-institutional collaborative. Urol Oncol. Dec 2021;39(12):836 e19–836 e27. doi:10.1016/j.urolonc.2021.08.016 34556430

[9] Haddad AQ, Wood CG, Abel EJ, Oncologic outcomes following surgical resection of renal cell carcinoma with inferior vena caval thrombus extending above the hepatic veins: a contemporary multicenter cohort. J Urol. Oct 2014;192(4):1050–6. doi:10.1016/j.juro.2014.03.111 24704115

[10] Padala SA, Barsouk A, Thandra KC, Epidemiology of Renal Cell Carcinoma. World J Oncol. Jun 2020;11(3):79–87. doi:10.14740/wjon1279 32494314 PMC7239575

[11] Neves RJ, Zincke H. Surgical treatment of renal cancer with vena cava extension. Br J Urol. May 1987;59(5):390–5. doi:10.1111/j.1464-410x.1987.tb04832.x 3594097

[12] Ciancio G, Vaidya A, Savoie M, Soloway M. Management of renal cell carcinoma with level III thrombus in the inferior vena cava. J Urol. Oct 2002;168(4 Pt 1):1374–7. doi:10.1097/01.ju.0000023441.00587.02 12352396

[13] Shah PH, Thompson RH, Boorjian SA, Symptomatic Venous Thromboembolism is Associated with Inferior Survival among Patients Undergoing Nephrectomy with Inferior Vena Cava Tumor Thrombectomy for Renal Cell Carcinoma. J Urol. Sep 2018;200(3):520–527. doi:10.1016/j.juro.2018.04.069 29709665

[14] Gonzalez J, Gaynor JJ, Martinez-Salamanca JI, Association of an organ transplant-based approach with a dramatic reduction in postoperative complications following radical nephrectomy and tumor thrombectomy in renal cell carcinoma. Eur J Surg Oncol. Oct 2019;45(10):1983–1992. doi:10.1016/j.ejso.2019.05.009 31155470 PMC8404534

[15] Boorjian SA, Sengupta S, Blute ML. Renal cell carcinoma: vena caval involvement. BJU Int. May 2007;99(5 Pt B):1239–44. doi:10.1111/j.1464-410X.2007.06826.x 17441917

[16] Lambert EH, Pierorazio PM, Shabsigh A, Olsson CA, Benson MC, McKiernan JM. Prognostic risk stratification and clinical outcomes in patients undergoing surgical treatment for renal cell carcinoma with vascular tumor thrombus. Urology. Jun 2007;69(6):1054–8. doi:10.1016/j.urology.2007.02.052 17572185

[17] Kaag MG, Toyen C, Russo P, Radical nephrectomy with vena caval thrombectomy: a contemporary experience. BJU Int. May 2011;107(9):1386–93. doi:10.1111/j.1464-410X.2010.09661.x 20883481 PMC4315148

[18] Wotkowicz C, Wszolek MF, Libertino JA. Resection of renal tumors invading the vena cava. Urol Clin North Am. Nov 2008;35(4):657–71; viii. doi:10.1016/j.ucl.2008.07.013 18992619

[19] Chen YH, Wu XR, Hu ZL, Treatment of renal cell carcinoma with a level III or level IV inferior vena cava thrombus using cardiopulmonary bypass and deep hypothermic circulatory arrest. World J Surg Oncol. Apr 22 2015;13:159. doi:10.1186/s12957-015-0584-8 25897659 PMC4411871

[20] Taweemonkongsap T, Nualyong C, Leewansangtong S, Surgical treatment of renal cell carcinoma with inferior vena cava thrombus: using liver mobilization technique to avoid cardiopulmonary bypass. Asian J Surg. Apr 2008;31(2):75–82. doi:10.1016/S1015-9584(08)60062-7 18490219

[21] Hevia V, Ciancio G, Gomez V, Alvarez S, Diez-Nicolas V, Burgos FJ. Surgical technique for the treatment of renal cell carcinoma with inferior vena cava tumor thrombus: tips, tricks and oncological results. Springerplus. 2016;5:132. doi:10.1186/s40064-016-1825-1 26933631 PMC4761352

[22] Ciancio G, Hawke C, Soloway M. The use of liver transplant techniques to aid in the surgical management of urological tumors. J Urol. Sep 2000;164(3 Pt 1):665–72. doi:10.1097/00005392-200009010-00012 10953122

[23] Zhang JP, Zhu Y, Liu YJ, Temporary filters and liver mobilization technique improve the safety and prognosis of radical nephrectomy and inferior vena cava thrombectomy in renal cell carcinoma with subdiaphragmatic thrombosis. Urol Int. 2013;91(3):279–84. doi:10.1159/000350521 23921190

[24] Blute ML, Leibovich BC, Lohse CM, Cheville JC, Zincke H. The Mayo Clinic experience with surgical management, complications and outcome for patients with renal cell carcinoma and venous tumour thrombus. BJU Int. Jul 2004;94(1):33–41. doi:10.1111/j.1464-410X.2004.04897.x 15217427

[25] Ciancio G, Gonzalez J, Shirodkar SP, Angulo JC, Soloway MS. Liver transplantation techniques for the surgical management of renal cell carcinoma with tumor thrombus in the inferior vena cava: step-by-step description. Eur Urol. Mar 2011;59(3):401–6. doi:10.1016/j.eururo.2010.07.028 20724064

[26] Tzakis A, Todo S, Starzl TE. Orthotopic liver transplantation with preservation of the inferior vena cava. Ann Surg. Nov 1989;210(5):649–52. doi:10.1097/00000658-198911000-00013 2818033 PMC1357802

[27] Parekh DJ, Cookson MS, Chapman W, Renal cell carcinoma with renal vein and inferior vena caval involvement: clinicopathological features, surgical techniques and outcomes. J Urol. Jun 2005;173(6):1897–902. doi:10.1097/01.ju.0000158459.42658.95 15879771

[28] Ciancio G, Shirodkar SP, Soloway MS, Livingstone AS, Barron M, Salerno TA. Renal carcinoma with supradiaphragmatic tumor thrombus: avoiding sternotomy and cardiopulmonary bypass. Ann Thorac Surg. Feb 2010;89(2):505–10. doi:10.1016/j.athoracsur.2009.11.025 20103332

[29] Abaza R, Eun DD, Gallucci M, Robotic Surgery for Renal Cell Carcinoma with Vena Caval Tumor Thrombus. Eur Urol Focus. Dec 15 2016;2(6):601–607. doi:10.1016/j.euf.2017.01.001 28723491

[30] Masic S, Smaldone MC. Robotic renal surgery for renal cell carcinoma with inferior vena cava thrombus. Transl Androl Urol. May 2021;10(5):2195–2198. doi:10.21037/tau.2019.06.15 34159102 PMC8185684

[31] Sun Y, de Castro Abreu AL, Gill IS. Robotic inferior vena cava thrombus surgery: novel strategies. Curr Opin Urol. Mar 2014;24(2):140–7. doi:10.1097/MOU.0000000000000033 24451090

[32] Campi R, Tellini R, Sessa F, Techniques and outcomes of minimally-invasive surgery for nonmetastatic renal cell carcinoma with inferior vena cava thrombosis: a systematic review of the literature. Minerva Urol Nefrol. Aug 2019;71(4):339–358. doi:10.23736/S0393-2249.19.03396-4 30957477

[33] Murphy C, Abaza R. Complex robotic nephrectomy and inferior vena cava tumor thrombectomy: an evolving landscape. Curr Opin Urol. Jan 2020;30(1):83–89. doi:10.1097/MOU.0000000000000690 31725003

[34] Abaza R Robotic surgery and minimally invasive management of renal tumors with vena caval extension. Curr Opin Urol. Mar 2011;21(2):104–9. doi:10.1097/MOU.0b013e32834350ff 21200324

[35] Wang B, Li H, Huang Q, Robot-assisted Retrohepatic Inferior Vena Cava Thrombectomy: First or Second Porta Hepatis as an Important Boundary Landmark. Eur Urol. Oct 2018;74(4):512–520. doi:10.1016/j.eururo.2017.11.017 29223604

[36] Wang B, Huang Q, Liu K, Robot-assisted Level III-IV Inferior Vena Cava Thrombectomy: Initial Series with Step-by-step Procedures and 1-yr Outcomes. Eur Urol. Jul 2020;78(1):77–86. doi:10.1016/j.eururo.2019.04.019 31103390

